# Assessment of plaque morphology in Alzheimer’s mouse cerebellum using three-dimensional X-ray phase-based virtual histology

**DOI:** 10.1038/s41598-020-68045-8

**Published:** 2020-07-08

**Authors:** Lorenzo Massimi, Nicola Pieroni, Laura Maugeri, Michela Fratini, Francesco Brun, Inna Bukreeva, Giulia Santamaria, Valentina Medici, Tino Emanuele Poloni, Claudia Balducci, Alessia Cedola

**Affiliations:** 1 Department of Medical Physics and Biomedical Engineering, University College London, London, UK; 2 Institute of Nanotechnology - CNR, Rome Unit, Rome, Italy; 3 Department of Anatomical Sciences, Histological, Legal Medical and Locomotor, University of Rome “Sapienza”, Rome, Italy; 4 IRCCS Santa Lucia Foundation, Rome, Italy; 5 Department of Engineering and Architecture, University of Trieste, Trieste, Italy; 6 Department of Neuroscience, Istituto di Ricerche Farmacologiche Mario Negri IRCCS, Milan, Italy; 7 Department of Neuropathology and Neurology, Golgi-Cenci Foundation, 20081 Abbiategrasso, Italy

**Keywords:** Diseases of the nervous system, X-ray tomography

## Abstract

Visualization and characterization of $\beta$-amyloid deposits is a fundamental task in pre-clinical study of Alzheimer’s disease (AD) to assess its evolution and monitor the efficiency of new therapeutic strategies. While the cerebellum is one of the brain areas most underestimated in the context of AD, renewed interest in cerebellar lesions has recently arisen as they may link to motor and cognitive alterations. Thus, we quantitatively investigated three-dimensional plaque morphology in the cerebellum in APP/PS1 transgenic mouse, as a model of AD. In order to obtain a complete high-resolution three-dimensional view of the investigated tissue, we exploited synchrotron X-ray phase contrast tomography (XPCT), providing virtual slices with histology-matching resolution. We found the formation of plaques elongated in shape, and with a specific orientation in space depending on the investigated region of the cerebellar cortex. Remarkably, a similar shape is observed in human cerebellum from demented patients. Our findings demonstrate the capability of XPCT in volumetric quantification, supporting the current knowledge about plaque morphology in the cerebellum and the fundamental role of the surrounding tissue in driving their evolution. A good correlation with the human neuropathology is also reported.

## Introduction

Alzheimer’s disease (AD) is the most common form of dementia affecting about 50 million people worldwide^1^. AD is characterized by progressive loss of memory and cognitive abilities causing severe interference in daily life of patients and is diagnosed, albeit not in a definitive manner, by careful examination of the patient based on live history, neurological exams, cognitive tests and brain imaging. Definitive diagnosis is achieved post-mortem when the two main histopathological hallmarks of the disease are revealed. They are represented by extracellular deposits of the $\beta$ amyloid peptide ($\beta$A) into the brain parenchyma and intracellular neurofibrillary tangles made of hyperphosphorylated tau protein^2^. $\beta$A deposits induce neuritic dystrophy and are considered as a reservoir of soluble $\beta$A species, namely oligomers, which are dynamic entities mainly responsible for synaptic and cognitive dysfunction, neuroinflammation, and neuronal cell death. Currently, PET imaging is the main technique for visualization of $\beta$A plaques in-vivo^3,4^. On the other hand, ex-vivo single plaque imaging presents challenges for standard investigation techniques. Histological sectioning provides good resolution and staining to unambiguously recognize $\beta$A plaques but slicing of the sample results in loss of volumetric information. Optical microscopy allows the recovery of volume information but is limited by low penetration of visible light. X-ray phase contrast tomography (XPCT) provides a valuable complementary tool overcoming many of the previous limitations using both synchrotron radiation and laboratory sources^5–7^. It provides contrast in soft tissues, down to the cellular level with minimum sample preparation and volumetric visualization^8–11^. Thanks to its capability to extract virtual slices of the tissue matching the histology resolution, XPCT is gaining importance in pre-clinical investigations as a 3D virtual histology technique, providing quantitative data also in pathological conditions^8,12,13^. In particular, accumulation of $\beta$A in mouse brain has been successfully imaged ex-vivo in animal models of AD without sample staining or sectioning^10,14,15^. Several transgenic mouse models have been developed in order to mimic AD pathology and facilitate the investigation of pathological mechanisms and therapeutic efficacy. These models are characterized by the presence of AD-related human mutations in the APP gene alone or in combination with mutations on the presenilin 1 or 2 genes, all involved in the release of the $\beta$A peptide. Thus, they develop a typical AD-related phenotype. In the present paper, we exploited XPCT to detect and quantitatively characterize plaque morphology inside the cerebellum of an APP/PS1dE9 mouse model of AD at advanced age. We focused on the plaque deposition in the cerebellum, since it remains one of the brain areas mostly underestimated in the context of AD with few histological observations reported both in AD patients^16–21^ and AD mice^22^. However, cerebellum involvement in AD has been attracting much attention recently, due to new evidence showing that cerebellar neuronal cell loss and synaptic alterations are linked to the onset of motor and cognitive alterations^23,24^. In addition, the cerebellar cortex presents a highly ordered structure both at macroscopic examination as well as at cellular level, providing an interesting base to explore the properties of plaques as a function of the surrounding tissue, and of the cytoarchitecture of that specific brain area. By exploiting the volumetric information provided by XPCT, plaque morphology as well as orientation in space has been assessed. We found the formation of plaques elongated in shape, with a log-normal distribution of the volume and with a specific orientation depending from the area of the cerebellum where they are localized. In particular, plaques in hemisphere and vermis are oriented mainly perpendicular to the transverse and sagittal planes, respectively. Our findings support the current knowledge about the morphology of AD plaque in cerebellum and the idea that surrounding tissue plays a fundamental role into their development. Moreover, a striking similarity with the plaques of the human cerebellum is found, suggesting that amyloid aggregates in humans develop through similar processes.

## Results

### Qualitative analysis

**Figure 1:**
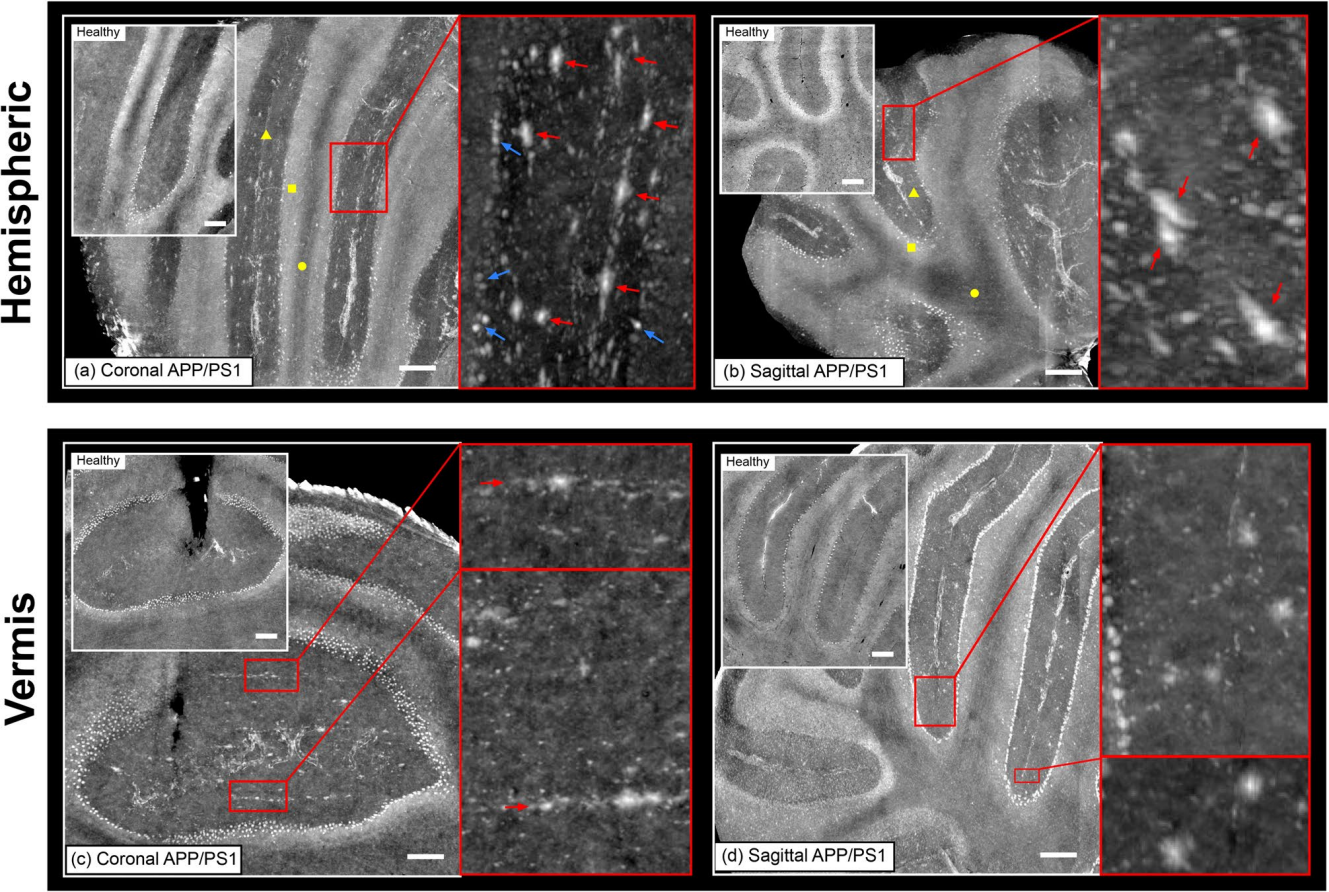
Cerebellar cortex in hemisphere and vermis of APP/PS1 mouse. Sagittal and coronal sections are shown in panels (**a**–**c**) and (**b**–**d**), respectively. Yellow triangle, square and circle in (**a**,**b**) indicate, molecular, granular and white matter layers, respectively. Interesting features are highlighted in the zoom-in panels. In particular, red arrows point at $\beta$A deposits and blue arrows at Purkinje cells located at the granular—molecular layer interface. Each XPCT section shown is obtained from maximum intensity projection (MIP) across 25 μm. Scale bars are 100 μm.

An APP/PS1 murine brain and an age-matched wild-type (WT) brain were compared by XPCT. The micron scale resolution achieved allows to distinguish $\beta$A plaques typical of the APP/PS1 AD mice, as well as to define sub-plaques details. To provide a qualitative description of $\beta$A plaques in the specimen a maximum intensity projection of tomographic slices across the main symmetry planes is shown in Fig. 1. Coronal (Fig. 1a,c) and sagittal (Fig. 1b,d) views of APP/PS1 cerebellum are shown together with the corresponding region of the healthy WT mouse brain (in the insets). According to the most common anatomical distinction, the cerebellum was divided in hemisphere and vermis.

The highly organized structure of the cerebellar cortex is distinguishable easily in both sagittal and coronal sections. Division in different layers of each folium is evident. Molecular, granular and white matter layers are all well distinguishable as per the difference in contrast and indicated respectively by yellow triangle, square and circle in Fig. 1a,b. In addition, hyper-intense spots are detected at the interface between granular and molecular layers, which are compatible in terms of size and position with Purkinje cells (blue arrows in zoom-in panels of Fig. 1a,d^25^. Compact and bright clusters located in the molecular layer are also seen, which are completely absent in the tissue of the WT mouse (see red arrows in zoom-in panels of Fig. 1a–d. These clusters are formed by a central dense core (represented in white grey scale) surrounded by a less dense corona as clearly seen in the zoom-in panels of Fig. 1a,b. The morphology is in agreement with $\beta$A plaques expected in APP/PS1 mice and already observed with XPCT at micron and nanometer resolution^10,14,15^. $\beta$A plaques are evenly spread all over hemispheric and vermal regions but they appear confined to the molecular layer. Moreover, plaques appear elongated in shape. In the hemisphere, such elongation is observed both in the sagittal and coronal sections, as pointed out by red arrows in zoom-in panels of Fig. 1a,b. Thus, plaque main axis is oriented perpendicularly with respect to the transverse plane. On the other hand, plaques in the vermis have a main axis perpendicularly oriented to the sagittal plane (see zoom-in panels of coronal and sagittal sections reported in Fig. 1c,d, respectively). In addition, plaques in the vermis appear arranged in parallel lines as highlighted by red arrows in the maximum intensity projection shown in the zoom-in panel of Fig. 1c. This finding suggests a volumetric arrangement in parallel, transversely oriented planes and has been observed also on other APP/PS1 cerebellum specimens (see Supplementary Image). Finally, no evidently defined arrangement was observed in plaques located in the hemisphere.

**Figure 2:**
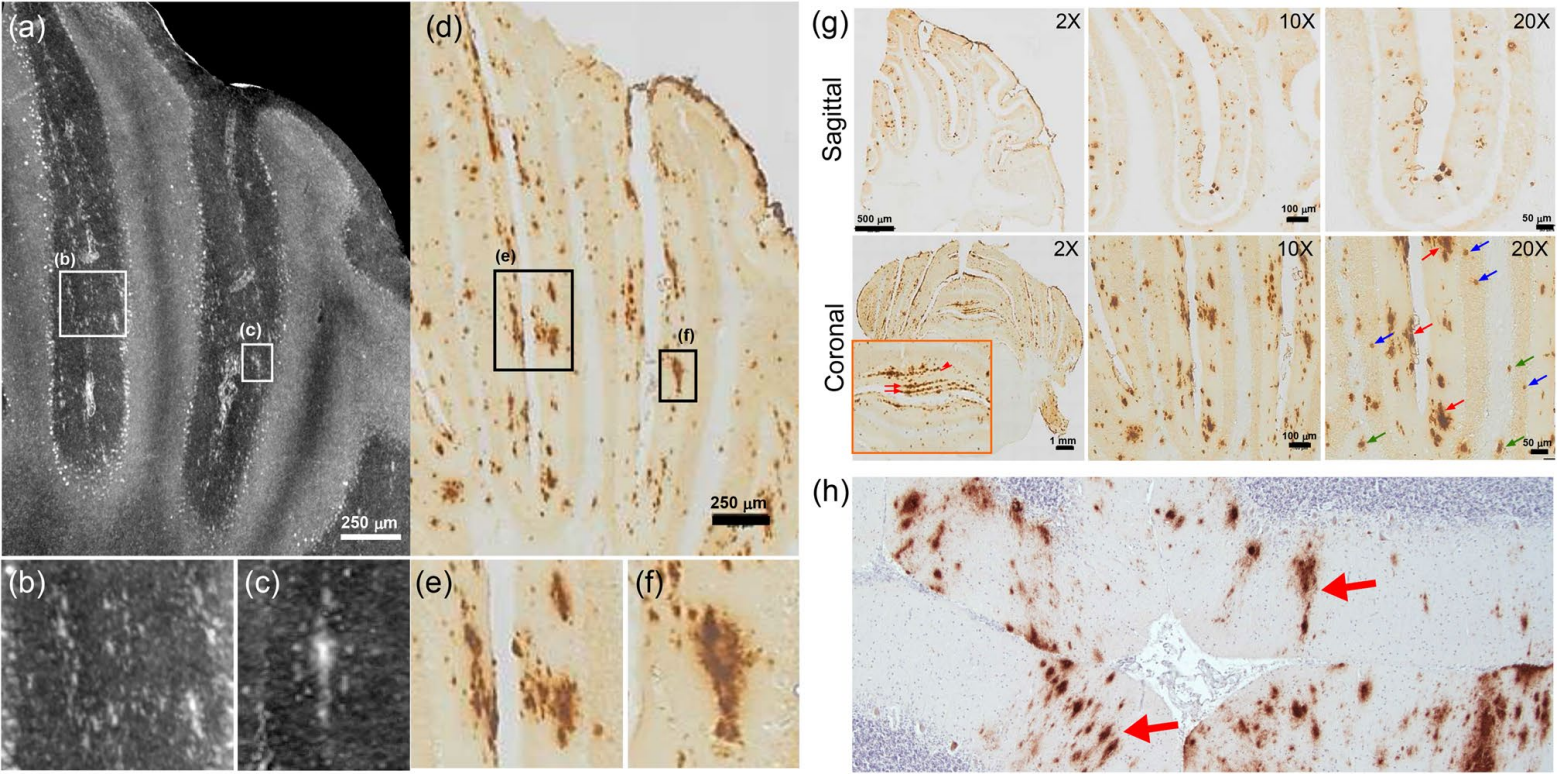
Comparison between XPCT and histology of cerebellar plaque deposition. (**a**–**c**) and (**d**–**f**) show a comparison between the best matched maximum intensity projection of tomographic slices (from hemisphere) and histology slices. (**g**) shows a set of stained sections in coronal and sagittal orientation. The inset in coronal 2 $\times$ panel shows the in-line arrangement of plaques in the molecular layer of the vermis detected in XPCT maximum intensity projection (red arrows). Red, blue, and green arrows in coronal 20 $\times$ panel point at plaques located in molecular and granular layers and white matter, respectively. Maximum intensity projection is obtained projecting voxel values across 20 μm in coronal direction. (**h**) shows a sagittal human cerebellar section stained with 4G8 antibodies showing both intracellular physiological amyloid into some Purkinje cells and pathological amyloid deposition into the molecular layer (4 $\times$). Red arrows highlight elongated shaped plaques resembling the shape of plaques observed in APP/PS1 mouse.

A comparison between a histological section and the best matched virtually selected XPCT slice is shown in Fig. 2a–f, respectively. A coronal and sagittal set of histological sections at different magnifications is also shown in Fig. 2g.

The comparison between the XPCT slices and the histological section confirmed the assignment of bright clusters detected in XPCT to $\beta$A deposits typical of AD (Fig. 2a,d). The elongated shape of plaques detected in the XPCT is corroborated by histology data (Fig. 2c,f). It is worth noting that this particular elongated shape, which definitely differs from the more roundish one typically found in the cortex and hippocampus, is also observed in the human cerebellum of patients with widespread amyloid deposition, reaching the fifth Thal’s phase, as shown by histology slice reported in Fig. 2h^26,27^. Examination of the histological sections (see Fig. 2g) confirms also the preferential location of plaques in the molecular layer, as already observed by XPCT both in vermis and hemisphere. However, histology reveals that plaques are also present in the granular layer, albeit of smaller size, lower in number and more round in shape. These plaques are indicated by blue (plaques in granular layer) and red (plaques in molecular layer) arrows in the coronal 20 $\times$ panel in Fig. 2g. In addition, an even lower number of small plaques appear located in the white matter (see green arrows in coronal 20 $\times$ panel in Fig. 2g). Finally, also the in-line arrangement of plaques observed in XPCT maximum intensity projection in the molecular layer of vermis is confirmed by immunohistochemistry, as shown by red arrows in the inset of the coronal $2\times$ panel in Fig. 2g pointing at three different lines of $\beta$A deposits.

### Volumetric quantification

**Figure 3:**
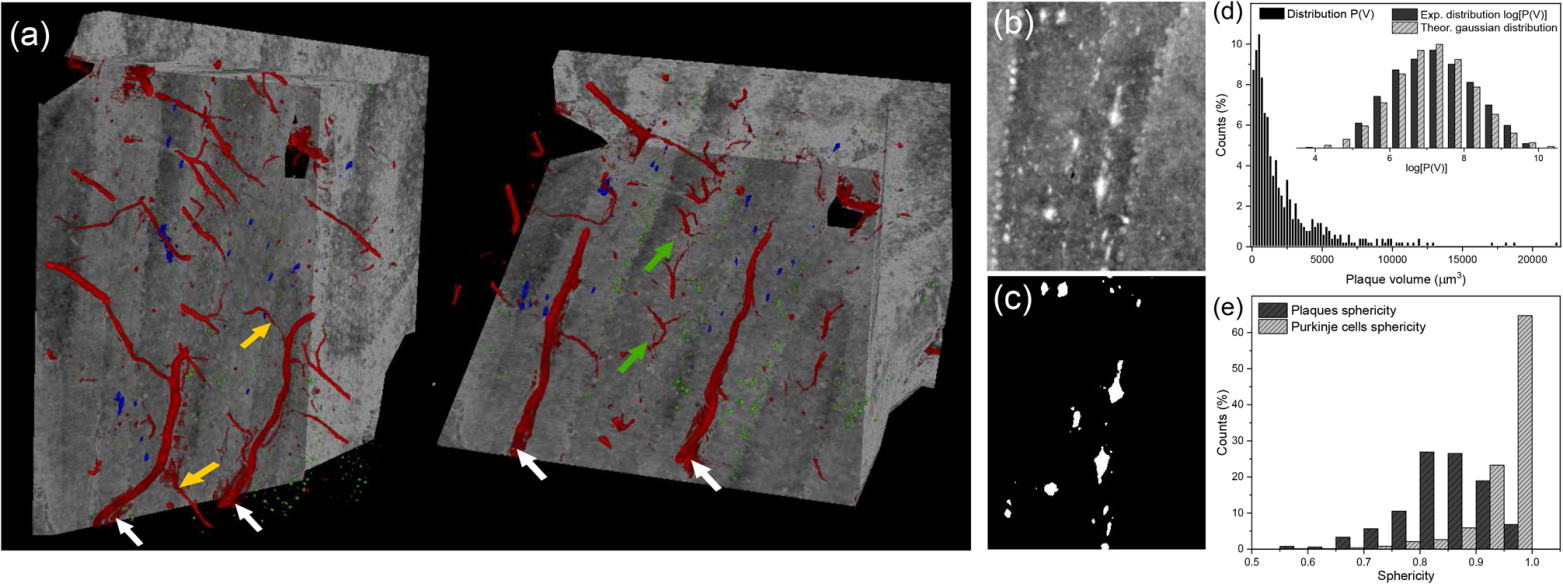
Quantification of plaque volume distribution and sphericity. (**a**) shows 3D renderings of a VOI of the APP/PS1 cerebellar cortex hemisphere. Plaques are rendered in blue, blood vessels in red and Purkinje cells in green. White arrows point at large blood vessels running in the coronal plane into the molecular layer. Yellow arrows point at branches of the large vessels oriented in the direction orthogonal to the coronal plane. Finally, green arrows highlight small blood vessels in the white matter oriented orthogonal to the coronal plane. (**b**,**c**) show a portion of maximum intensity projection of cerebellar tissue in the hemisphere region and the binary mask originated by segmentation of plaques in the same region, respectively. (**d**) shows the histogram distribution of plaque volume P(V). In addition, in the inset a comparison between the distribution of log(P(V)) and a gaussian distribution with the same standard deviation and mean value is shown, proving that P(V) is log-normal distributed. For the sake of comparison, both distributions are normalized to the area. The graph in (**e**) shows the histogram distribution of sphericity for both plaques and Purkinje cells.

XPCT measurement has the unique advantage of providing volumetric information on micron and sub-micron scales. Thus, it allows the calculation of 3D properties otherwise impossible with standard techniques. By exploiting this 3D capability, we could virtually extract plaques from volumes of interest located both in hemisphere and vermis, and characterize their properties, such as volume, sphericity, and distribution of the orientation in space. The results are shown in Fig. 3a–e.

3D renderings of a volume of interest (VOI) in the hemisphere cortex allow a better view on plaque morphology and their arrangement in space. In particular, plaques, marked in blue, are easily detected and their elongated shape appears evident in the top rendering of Fig. 3a. Purkinje cells appear as small spheres, in green, lined at the interface between granular and molecular layers (see bottom rendering of Fig. 3a). Blood vessels are also observed and rendered in red (see both panels of Fig. 3a. A partitioning of blood vessels is found, which follows the high ordered structure of the cerebellar cortex. Large blood vessels lie in coronal plane and run along the molecular layer (see white arrows in panels in Fig. 3a). Smaller branches depart from them running orthogonal to the molecular layer. On the other hand, small blood vessels orthogonal to the coronal plane are found in the white matter, as pointed out by green arrows in the lower panel of Fig. 3a (see also Supplementary Data for blood vessels arrangement). In order to quantify the volume and elongated shape qualitatively observed in XPCT slices and histology, plaques have been extracted by means of a semiautomatic segmentation approach based on gray level thresholding^8^. Purkinje cells have also been segmented^25^. An exemplary mask obtained from segmentation of plaques in XPCT volumes is shown in Fig. 3c in comparison with the corresponding XPCT section in Fig. 3b, revealing a good match between segmented features and $\beta$A plaques detected in the slice. From the segmented binary mask, features properties can then be easily calculated. Histogram distribution of plaques volume P(V) is reported in Fig. 3d. No overall difference between plaques in hemispheric and vermal regions was found in terms of volume. Thus, plaques from both regions have been included in the P(V) distribution. Volume distribution P(V) shows that 50% of the segmented plaques have a volume smaller than about $10^3$ μm^3^, while 90% are included increasing the volume threshold up to $5\times 10^3$ μm^3^. However, plaques up to $20\times 10^3$ μm^3^ are found. Moreover, P(V) shows a log-normal distribution, as shown by the plot of log(P(V)) in comparison with a discrete gaussian distribution with the same mean and standard deviation (see the inset of Fig. 3d). In addition to characterization of volume distribution, we exploited segmented plaques to quantify the elongation in shape qualitatively already observed in XPCT and histology. For this task, sphericity (hereafter indicated with S) was calculated. The data from around 500 plaques is shown in Fig. 3e. Remarkably, the sphericity shows a peaked distribution with a mean value of $S_{\mu }$ = 0.82 and standard deviation $\sigma$ = 0.07. Moreover, a longer tail towards low sphericity values is observed and compatible with highly elongated plaques. A negative skewness (− 0.78) confirms the asymmetry. In comparison, Purkinje cells show an average sphericity value of S = 0.94 and standard deviation $\sigma$ = 0.05. Purkinje cells sphericity is significantly higher $(P < 0.05)$ than $\beta$A plaques (see Fig.3e). No overall difference between sphericity of plaques located in hemisphere or vermis is found.

**Figure 4:**
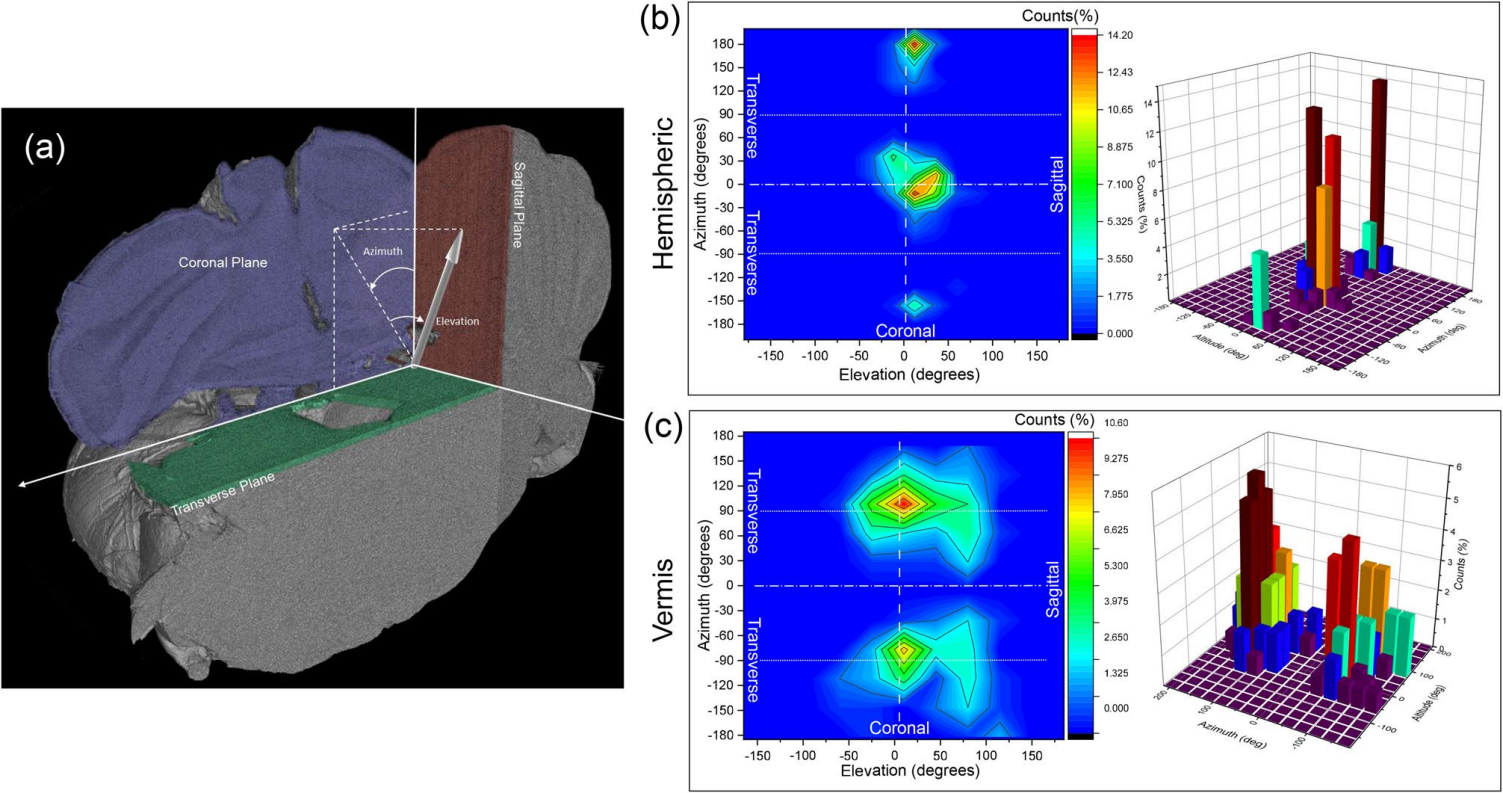
Plaque orientation in hemispheric and vermal regions. (**a**) shows the orientation of the reference system with respect to the tomographic volume. (**b**,**c**) show contour plot and 3D histogram of the distribution of plaque orientation in terms of azimuth and elevation in hemisphere and vermis, respectively. Data are distributed in the $\pm \,180^{\circ }$ range for both azimuth and elevation.

The low average sphericity found for $\beta$A deposits has been exploited to characterize their orientation in space and to quantitatively assess the different orientation qualitatively observed in hemisphere and vermis. Orientation of each plaque in space has been calculated as the orientation of main axis of an ellipsoid containing the plaque itself and described in terms of azimuth and elevation angles (see reference system in Fig. 4a). Their distribution is reported in a 3D histogram and a 2D contour plot matrix in Fig. 4b,c.

In hemisphere, plaques oriented almost perpendicularly to transverse plane have been qualitatively observed. Remarkably, this behavior is confirmed by quantitative analysis. According with the scheme shown in Fig. 4a, a combination of elevation and azimuth identifies the main symmetry planes indicated by lines in the contour plots. In particular, coronal plane is characterized by elevation equal to $0^{\circ },\,\pm\,180^{\circ }$ for all azimuth. It is worth noting that the three angles $0^{\circ },\,\pm\,180^{\circ }$ indicate the same orientation since no direction can be defined for the plaque. The distribution appears clustered around $0^{\circ },\,\pm\,180^{\circ }$ in azimuth and around $0^{\circ }$ in elevation, corresponding to a direction normal to transverse plane and lying in both coronal and sagittal planes. Moreover, the deviation from coronal and sagittal planes is indicated from width of data distribution in elevation and azimuth, respectively. In hemisphere, a deviation from both the coronal and sagittal plane less than $30^{\circ }$ is found. Thus, plaques are oriented within a cone with main axis normal to transverse plane and with a semi-angle of about $30^{\circ }$. Remarkably, the observed spread appears in agreement with change in orientation of the molecular layer moving through the folium in the volume of interest (see Supplementary Data). The orientation observed in the vermis, with plaques oriented almost in the transverse plane, is also confirmed by quantitative analysis as shown Fig. 4c. Contour plot and 3D histogram clearly show accumulation around azimuth value $\pm\, 90^{\circ }$ ($\pm\, 90^{\circ }$ indicate the same orientation). This is compatible with the orientation of a vector in transverse plane characterized by azimuth $\pm 90^{\circ }$ for any elevation. A deviation from the coronal plane of about $60^{\circ }$ is found in contrast with a narrower deviation from the transverse plane of about $30^{\circ }$. Thus, plaques direction in vermis lies in an asymmetric cone with main axis normal to sagittal plane and a semi-angle of $60^{\circ }$ in the transverse plane (deviation from coronal plane) and less than $30^{\circ }$ in the coronal plane (deviation from transverse plane). It is worth noting that a systematic shift from high symmetry planes is observed in both distributions i.e. in hemisphere and vermis. This difference may be due to a misalignment of the tomographic volume with respect to the real symmetry planes of the sample.

## Discussion

The presence of deposits of $\beta$A protein is considered one of the main hallmarks of AD. In addition, amyloid may probably induce other proteinopathies^28^. There is a large interest in imaging of $\beta$A plaques especially while evaluating the ability of new therapeutic approaches to induce plaque reduction/elimination^29^. Plaques are easily detected in phase contrast volumes of murine cerebellum as hyper-intense clusters. In particular, plaques with a dense core surrounded by a corona are observed^10,30,31^. The higher density of the core may be explained considering the presence of metals inside the plaque^31,32^. Remarkably, inspection of XPCT slices reveals that plaques are concentrated in molecular layer while they are not observed in others layers of the cerebellar cortex. This has been confirmed by histology showing, in addition, few smaller plaques hardly detectable with XPCT in both granular layer and white matter. The difference with XPCT findings can be explained considering the higher grey level value of granular layer in XPCT compared to the molecular one. Thus, a similar grey value hinders plaque detection in the granular layer. Furthermore, plaques in granular layer may be mistaken with Purkinje cells when located at the interface with molecular layer due to similar size and grey value. Differences in the prevalence of plaques between the granular and molecular layer might be related to differences in the structural layers of the cerebellum related to the numbers and size of neuronal cell bodies, axons, dendrites, and terminals^33^. Irrespective of the origin of amyloid protein in AD brain, the morphological diversity of the deposits could conceivably be related to differences in the size of neurons and/or their processes^33^. Moreover, the smaller size of plaques outside the molecular layer might be related to a different timing in plaque evolution. It could be speculated that $\beta$A deposits may occur in granular layer and white matter in an advanced stage after that they spread all over the molecular layer. Plaques appear with an elongated shape and oriented in a preferential direction. In hemisphere they are oriented normal to transverse plane following the direction of cerebellar cortex, whereas in the vermis they are oriented normal to the sagittal plane forming aligned clusters. Both shape and line clustering have been confirmed by histology. This finding is in agreement with histological observations of similar elongated plaques in human cerebellum, thus suggesting that the anatomy and cytoarchitecture of that specific brain area might dictate plaque organization and distribution^16,17^. To our best knowledge, no observation of such a peculiar shape is reported for other regions of the brain, suggesting a fundamental role in plaque development of the highly symmetric structure of the cerebellar cortex. Segmentation of plaques has been a fundamental step to support our qualitatively observations and to fully exploit the volumetric information provided by XPCT. In this work, a semi-automatic approach has been used involving human intervention to select a region of interest around plaques and then segmenting with an intensity threshold. This approach has the disadvantage of human intervention being time consuming and thus limiting its application to small volumes. Moreover, Purkinje cells at the boundary of granular and molecular layers were also segmented in order to provide a comparison in sphericity with respect to plaques. In this case a shape-based segmentation approach has been used based on the almost perfect round shape of the cellular body^25,34^. Despite the highlighted limitations, the segmentation procedure adopted in this work allowed to extract hundreds of plaques providing a reliable statistic on plaque volume and sphericity. We found a log-normal distribution P(V) of plaque volume. In general, a log-normal distribution of clusters size is frequently found in nucleation and growth processes and it is compatible with a growing rate proportional to cluster size itself^35,36^. Remarkably, a log-normal distribution has been also specifically reported for the distribution of surface area of senile human Alzheimer’s plaques in cerebral cortex determined through histology and it agrees with a model where plaque growing rate is proportional to the plaque volume^37–39^. An increase in growing rate proportionally to the volume supports a structural model of the plaque as a non-compact aggregate where growing process is not limited to the surface, in agreement with other findings^10,37,38,40^. This observation also correlates with a low PET signal found in the cerebellum and explained by diffuse nature of $\beta$A deposits^41^. Moreover, the peculiar elongated shape of plaques, confirmed by the distribution of sphericity, suggests that a strong constraint in plaque growth is imposed by the surrounding tissue. Supporting this hypothesis, we observed that plaques are aligned in different directions in hemisphere and vermis. In the former, they are normal to transverse plane, while in the latter they are almost normal to sagittal plane, despite with a less strict distribution. Thus, a change in tissue arrangement is reflected in a change of plaque orientation. This observation supports the idea that plaque evolution is driven by the surrounding tissue^21^. In particular, the compact and ordered arrangement of the cerebellar tissue may physically limit the available space forcing plaques to grow along a specific direction. Further studies are currently on going to investigate plaque morphology also in different brain regions, exploiting 3D visualization provided by XPCT.

## Methods

### Mice

The APPswe/PS1$\Delta$e9 (APP/PS1) transgenic male mouse [B6C3-Tg(APPswe,PSEN1dE9)85Dbo/Mmjax] and the WT littermate used for this experiment were purchased from Jackson Laboratories (USA). No environmental enrichment was used since it notably improves AD pathology in mouse models of AD^42,43^. Mice were all drug and behavioral test naive and the experiments were all conducted during the light cycle. All animals were housed in a SPF facility in groups of 4 in standard mouse cages containing sawdust with food (2018S Harlan diet) and water ad libitum, under conventional laboratory conditions (room temperature: 20 ± 2 °C; humidity: 60%) and a 12/12 hour light/dark cycle (7:00 am–7:00 pm). The research protocols were reviewed and approved by the Mario Negri Institute Animal Care and Use Committee, that includes members specific for ethical issues, and the Italian Ministry of Health. The Mario Negri Institute for Pharmacological Research adheres to the principles set out in the following laws, regulation, and policies governing the Care and Use of Laboratory Animals: Italian Governing Law (D.lgs 26/2014; Authorization n.19/2008-A issued March 6, 2008 by Ministry of Health); Mario Negri Institutional Regulations and Policies providing internal authorization for persons conducting animal experiments (Quality Management System Certificate—UNI EN ISO 9001:2015—Reg. No 6121); the NIH Guide for the Care and Use of Laboratory Animals (2011 edition) and EU directives and guidelines (EEC Council Directive 2010/63/UE). The statement of Compliance (Assurance) with the Public Health Service (PHS) Policy on Human Care and Use of Laboratory Animals has been reviewed (9/9/2014) and will expire on September 30, 2019 (Animal Welfare Assurance #A5023-01).

### Sample preparation

One mouse for each condition (APP/PS1 and WT) has been used for XPCT experiments. Both mice were killed under CO_2_ to drastically reduce bleeding. Brains were removed, placed in paraformaldehyde for 24 h, and stored in 70% EtOH at 4 °C until analysis. After XPCT data acquisition the brains of the wild-type and APP/PS1 mice were sectioned along both coronal and sagittal planes (20 μm thick) and incubated for 1 h at room temperature (RT) with 10% normal goat serum (NGS) then overnight (O/N) at 4 °C with primary antibodies (6E10 1:500; Signet). After incubation with the appropriate biotinylated secondary antibodies (1:200; Vector Laboratories), immunostaining was developed using the avidin-biotin kit (Vector Laboratories) and diaminobenzidine (Sigma, Italy) as chromogen.

### Human subject

For a comparison with human histopathology, a case of severe amyloid deposition was selected from the Abbiategrasso Brain Bank (ABB) series. The histology section comes from a case of 84-year-old woman who died with a clinical diagnosis of Major-NCD(dementia) due to probable Azheimer’s Disease. She presented a typical clinical onset with memory impairment and progressive involvement of other cognitive domains. During the intermediate phase of the disease, she also manifested behavioral and psychotic symptoms. Neuropathological examination revealed widespread amyloid deposition corresponding to a Thal 5 stage (diffuse cortical, brainstem and cerebellar involvement), associated with meningeal amyloid angiopathy, intermediate Alzheimer TAU pathology (Braak IV TAUopathy), limbic sinucleinopathy and limbic TDP-43 deposition and moderate basal ganglia small vessel disease. The neuropathological definite diagnosis of this case is dementia due to multiple aetiologies with severe and widespread amyloid deposition (Thal 5). In line with our institution’s Human Research Ethics Committee and the BrainNet Europe’s Code of Conduct, the ABB performs its activities following ethical standards^44,45^. The brain harvesting procedure was submitted to and approved by the Ethics Committee of the University of Pavia in the context of the InveCe.Ab study^46^. Joining the donation program is a personal decision, and complete awareness is needed.

### XPCT data acquisition

Experimental tomography datasets from APP/PS1 and wild-type mice were collected at the TOMCAT beamline of the Swiss Light Source (SLS), in Villigen, Switzerland^30^. Phase contrast tomography measurement was performed with 17 keV monochromatic radiation in free space propagation mode with a propagation distance of 5 cm. Images were detected with a PCO edge camera (sensor size 2560 $\times$2160 pixels and 6.5 μm pixel size) coupled with a scintillator screen and 4 $\times$ optic. The effective pixel size was 1.625 μm resulting in a field of view of 4.2 $\times$3.5 mm^2^. Tomography was performed acquiring 3000 projections in half acquisition mode almost doubling the native field of view. Sample-detector distance has been chosen according to the beam energy and the pixel size^47,48^. During acquisition sample was kept in air and reconstruction plane was almost coincident with coronal plane. Data reconstruction has been performed with SYRMEP Tomo Project software implementing ASTRA toolbox for efficient tomographic reconstruction on GPU^49,50^. Filter back projection algorithm has been used. Ring artifacts have been suppressed by means of an improved frequency filtering implemented in Matlab^51^.

### Volumetric quantification

Quantification of volumetric data was performed with custom routines developed in Matlab^52^. Segmentation process is composed of two steps. The first requires user intervention. Exploiting the projection of maximum intensity value of a small number of slices plaques are easily identified by visual inspection and a circular ROI is created around it. This procedure avoids creating large ROIs also for small plaques increasing the risk of including other features from the tissue and blood vessels. The procedure is then repeated for each plaque and each group of projected slices. Thus, cylindrical VOIs with different diameters and height depending the number of projected slices are created around each identified plaque. Finally, the histogram of grey values of voxels included in all VOIs is calculated. In the histogram two different peaks can be easily detected and assigned to background of the tissue and plaque, respectively. Then, an intensity-based segmentation extracts binary mask of plaques^8^. We note that even if a small blood vessel is found in the extracted VOIs, the large difference in brightness compared to plaques prevents its inclusion in the segmented features (see Supplementary Data). In addition, only segmented structures with a volume higher than 150 μm^3^ have been considered. This threshold avoids the inclusion of small structures not compatible with plaques that could be erroneously included in the segmentation. Around 500 features fulfilling the $>150$ μm^3^ inclusion criterion, and thus compatible with plaques, were found and used for calculation of volume distribution and sphericity. Purkinje cells have been segmented exploiting their almost perfect spherical shape detected at this resolution. A modified Frangi’s filter approach based on quantification of eigenvalues of Hessian matrix has been used^25,34,53^. The obtained binary masks have been used to extract volume (plaques) and sphericity (plaques and Purkinje cells) of each binary connected region. The sphericity is calculated as the ratio between the surface area of a sphere with the same volume of the given binary region and the surface of the region itself. A perfect sphere has $S=1$ while elongated structures have $S<1$. In order to calculate orientation of $\beta$A plaques in space only plaques with sphericity in the range $S < S_{\mu }+\sigma$ have been considered, where $S_{\mu }$ and $\sigma$ are the mean value and standard deviation of plaques sphericity distribution. This avoids including objects too close to a sphere for which orientation is not well defined. Thus, 200 and 260 plaques fulfilling the inclusion criterion defined above have been found in hemispheric and vermal regions, respectively. Then, eigenvectors and eigenvalues have been calculated. Orientation of the eigenvector with higher eigenvalue has been considered as orientation of the plaque in space and reported as azimuth and elevation in a contour plot and 3D histogram. Graphs and contour plot have been made with OriginPro^54^. Final images have been created with ImageJ and Inkscape^55,56^.

## Supplementary information

Supplementary Information

## Data Availability

Data availability. The authors declare that the data supporting the findings of this study are available from the corresponding author on reasonable request.
