# Supplementary material for: Assessment of plaque morphology in Alzheimer’s mouse cerebellum using three-dimensional X-ray phase-based virtual histology
Source: Sci Rep. 2020 Jul 8;10:11233. doi: 10.1038/s41598-020-68045-8 (PMC7343834; doi:10.1038/s41598-020-68045-8)
Supplement: Supplementary file 1 — Supplementary Information [file 41598_2020_68045_MOESM1_ESM.pdf]

Assessment of plaque morphology in Alzheimer's mouse cerebellum using three-dimensional X-ray phase-based virtual histology: supplementary image and data

Lorenzo Massimi[1,2,*], Nicola Pieroni[2,3], Laura Maugeri[2,4], Michela Fratini[2,4], Francesco Brun[2,5], Inna Bukreeva[2], Giulia Santamaria[6], Valentina Medici[7], Tino Emanuele Poloni[7], Claudia Balducci[6], and Alessia Cedola[2]

[1]Department of Medical Physics and Biomedical Engineering, University College London, London, UK
[2]Institute of Nanotechnology - CNR, Rome Unit, Rome, Italy
[3]Department of Anatomical Sciences, Histological, Legal Medical and Locomotor, University of Rome "Sapienza", Rome, Italy
[4]IRCCS Santa Lucia Foundation, Rome, Italy
[5]Department of Engineering and Architecture, University of Trieste, Trieste, Italy
[6]Department of Neuroscience, Istituto di Ricerche Farmacologiche Mario Negri IRCCS Milan, Italy
[7]Department of Neuropathology and Neurology, Golgi-Cenci Foundation, 20081 Abbiategrasso, Italy
[*]l.massimiphd@gmail.com

We performed other experiments on APP/PS1 mouse model using a different experimental setup. Fig. S1 shows a maximum intensity projection along the coronal direction of tomographic slices of the vermis region of an APP/PS1 mouse cerebellum. The different layers of cerebellar cortex are easily detected: molecular, granular and white matter layers (yellow triangle, square and circle in Fig. S1, respectively). Purkinje cells layer is also easily resolved (yellow arrow in Fig. S1). Large s-shaped blood vessels are also visible in the molecular layer (see blue arrows in Fig. S1). bA plaques appear as dense spots, indicated by red arrows. However, the achieved spatial resolution does not allow to resolve the difference between the central dense core and less dense corona observed at higher spatial resolution. Also, in this specimen, plaques in vermis appear arranged on parallel transverse planes and spread across the molecular layer only, supporting the findings reported in the main manuscript.

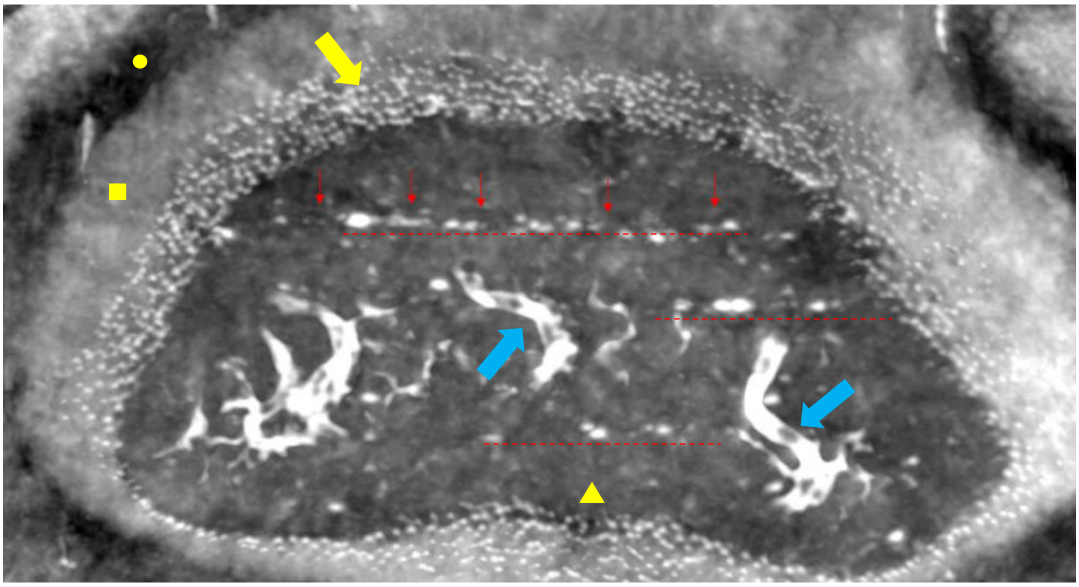

Figure S1. Coronal section of cerebellar cortex in vermis of APP/PS1 mouse. Red arrows mark plaques while the red dotted lines mark aligned cluster of plaques. Yellow, circle, square, triangle and arrow point at white matter, granular layer, molecular layer and Purkinje cells layer, respectively. Blue arrows point at blood vessels.

The supplementary experimental dataset has been obtained at the ID17 beamline of European Synchrotron Radiation Facility (ESRF), in Grenoble (France) using free space phase contrast mode with a propagation distance of 2.3 meters and X-ray energy of 35 keV (monochromatic). Tomography has been performed acquiring 3600 projections in half acquisition mode. Projections have been collected by a PCO.Edge 5.5 sCMOS camera coupled with a scintillator screen and a 2x lens allowing a final effective pixel size of 3.1 µm.

In Fig.S2 more details about plaque orientation in the hemisphere region are given.

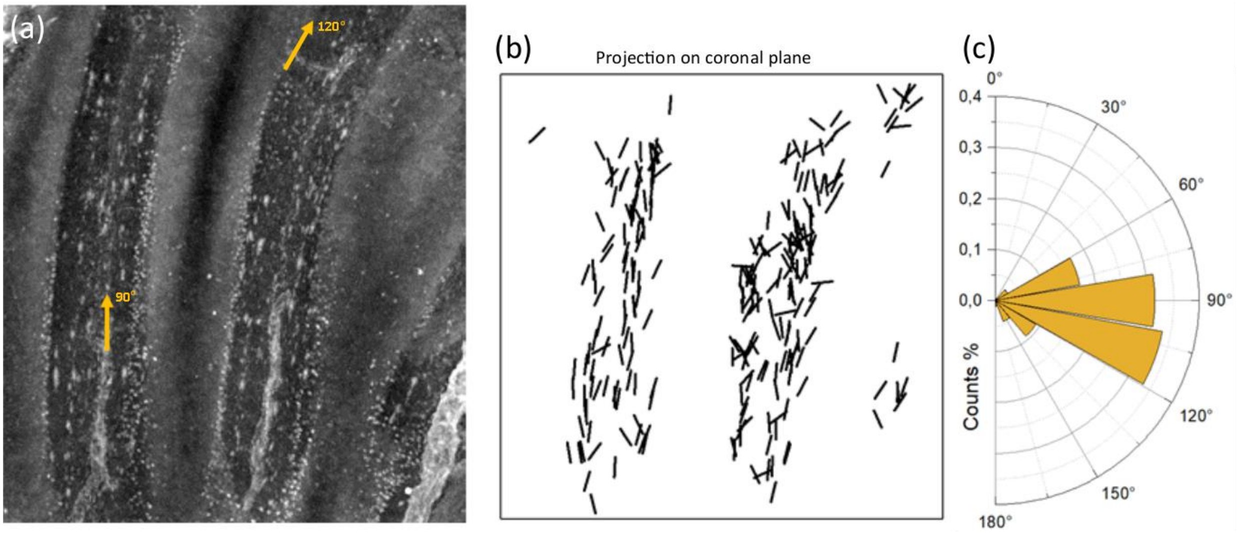

Figure S2. Panel a shows a maximum intensity projection of a portion of the investigated hemispheric volume. Panel b shows the projection of major axis of the segmented plaques on the coronal plane. Finally, panel c reports the angular distribution of the major axis projection of plaques shown in the previous panel.

In particular, the maximum intensity projection of a portion of the investigated volume of interest in the hemisphere shows the change in orientation of the folium, from a straight direction (90°) to a tilt of about 30° (see arrows in FigS2a). In addition, in Fig.S2b the projections on the coronal plane of the major axis of the segmented plaques is shown and combined with their angular distribution (reported in Fig.S2c). The latter, reported in Fig.S2c, clearly shows the angular spread of about ±30°. Remarkably, the distribution appears asymmetric with a clustering around +30°, in agreement with the change in the orientation of cerebellar folium shown in panel (a). These data show a change in plaque orientation also within the same cerebellar cortex region, where changes in tissue arrangement are supposed to be less drastic and may also explain the angular spread observed in the angular distribution reported in Fig.4 of the main manuscript.

In fig. S3 comparison between plaque and vessel segmentation is shown to illustrate spatial relation between segmented plaque and vascular system and to demonstrate no blood vessels features have been included in plaque statistic. It worth noting that segmentation has been performed in the volume, but the maximum

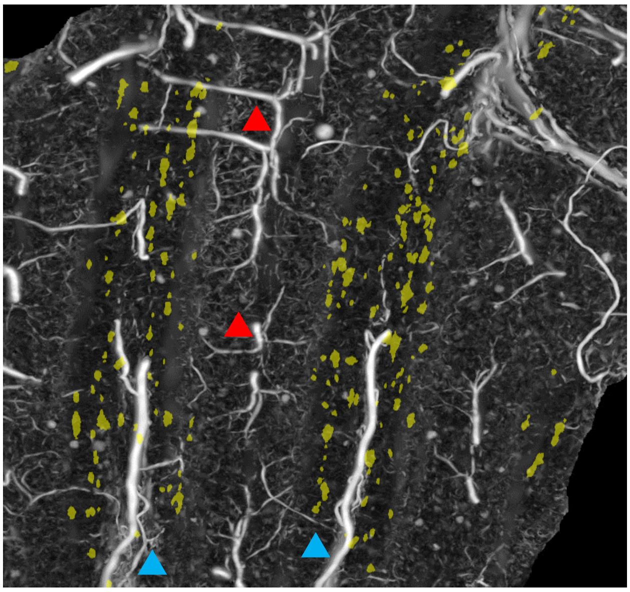

Figure S1. Overlap of segmented plaques (yellow) and segmented blood vessels.

intensity projection is shown here. The arrangement of blood vessels already discussed in the main manuscript as comment to the 3D renderings is well visible. Two large vessels (see light blue triangles) are

running within the molecular layer while an ordered network of smaller vessels is visible in the white matter (see red triangles). In addition, Fig.S3 reveals that, while some blood vessels appear hyperintense in the maximum intensity projection (see blue arrows in Fig.S1) with a contrast similar to plaques, the procedure used to isolate plaques prevented inclusion of such a features into the segmented objects. Blood vessels segmentation shown here has been performed by means of the Frangi's filter *(see Frangi, Alejandro F., et al. "Multiscale vessel enhancement filtering." International conference on medical image computing and computer-assisted intervention. Springer, Berlin, Heidelberg, 1998)* that is specifically designed to extract tubular structures regardless of their intensity. The brighter the pixel the more likely it belongs to a tubular structure (blood vessel).
